# Direct Shear Stress Mapping Using a Gallium Nitride LED-Based Tactile Sensor

**DOI:** 10.3390/mi14050916

**Published:** 2023-04-24

**Authors:** Nathan Dvořák, Nima Fazeli, Pei-Cheng Ku

**Affiliations:** 1 Department of Electrical Engineering and Computer Science, University of Michigan, 1301 Beal Ave., Ann Arbor, MI 48109-2122, USA; nadvorak@umich.edu; 2 Department of Robotics, University of Michigan, 2505 Hayward St., Ann Arbor, MI 48109-2122, USA; nfz@umich.edu

**Keywords:** piezoelectric effect, robotics, slip detection

## Abstract

An experiment was performed to calibrate the capability of a tactile sensor, which is based on gallium nitride (GaN) nanopillars, to measure the absolute magnitude and direction of an applied shear force without the need for any post-processing of data. The force’s magnitude was deduced from monitoring the nanopillars’ light emission intensity. Calibration of the tactile sensor used a commercial force/torque (F/T) sensor. Numerical simulations were carried out to translate the F/T sensor’s reading to the shear force applied to each nanopillar’s tip. The results confirmed the direct measurement of shear stress from 3.71 to 50 kPa, which is in the range of interest for completing robotic tasks such as grasping, pose estimation, and item discovery.

## 1. Introduction

Sensing and mapping a low-magnitude shear force field, including both the force’s magnitude and direction, are essential to monitoring and controlling grasped objects [1]. Studies have shown that humans modulate their grasp forces to maintain a margin above the minimum threshold required to prevent slip [2,3]. Slip occurs when the applied shear force by the contacts is insufficient to balance out external forces (e.g., gravity and/or external contacts) applied to the object. Humans can monitor localized slip and shear forces using mechanoreceptors at the fingertips and modulate grasping forces to ensure stable grasps [2]. Monitoring and control are primarily subconscious and adaptive to various external factors, such as changes in physical properties (e.g., the addition of dirt or liquids) and external forces. Unfortunately, robotic tactile sensors and systems are currently limited in this functionality. Instead, modern tactile sensing solutions focus on measuring imprints made due to contact with deformable media [4,5,6,7]. These measurements are made using RGB or depth cameras and have proven effective for pose estimation and extrinsic contact detection for the grasped object [8,9,10]. However, to convert these vision-based signatures to forces, inverse Finite Element Methods (FEMs) need to be employed [11] which can be computationally expensive and challenging to tune. Prior work has focused on detecting slip using a purely geometric approach (i.e., sensor gel deformation) without computing forces [12]. This approach relies on a rigidity assumption and careful thresholding that can be difficult to tune in practice. Further, it cannot estimate external forces applied to the grasped object without additional sensing apparatus.

Other MEMS (micro-electromechanical systems)-based tactile sensors deduce applied forces by monitoring changes in piezo resistance and capacitance [13,14,15,16,17,18,19,20,21,22,23]. Scaling these sensors for a large area (>1 mm^2^) while maintaining high spatial-resolution sensing requires high-quality fabrication control, thus increasing sensor cost. In recent years, optical methods for tactile sensing have emerged [24,25,26,27]. These sensors do not require high material uniformity for large-scale tactile sensing and have a customizable sensing resolution.

This paper presents the direct shear force measurement and mapping, including the force’s magnitude and direction without any post-data processing required, using a gallium nitride (GaN) nanopillar light-emitting diode (LED)-based tactile sensor. We also show that the sensor can detect the oscillation between the slip and grasp states.

## 2. Materials and Methods

**Figure 1:**
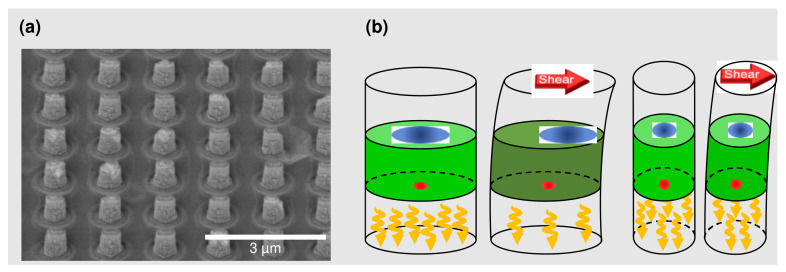
(**a**) Scanning electron micrograph (SEM) of an array of nanopillar-shaped light-emitting diodes. Each nanopillar has an elliptically shaped cross-section. Directional tactile sensing is achieved with two groups of nanopillar LEDs with their ellipses orthogonally oriented (the second orientation is not shown in the SEM image here). The height-to-width ratios are 2 and 4 when measured from the long and short axes, respectively. The nanopillar LEDs can be optically [25] or electrically biased [26]. (**b**) Illustration of the operating principle of the tactile sensor. The shear force applied to the tip of the nanopillar deforms the nanopillar and shifts the electron (blue) and hole (red) wavefunctions. Holes remain nearly unmoved at the center due to their large effective mass. Electrons move against the direction of the force. When the electron and hole wavefunctions are misaligned, the emission intensity is reduced. Monitoring the relative change of the emission intensity allows one to measure the force’s magnitude. The nanopillar’s finite dimension limits the travel of the electrons. The elliptically shaped cross-section leads to a different number of travels for the electrons. The difference in emission intensity allows us to determine the force’s direction.

**Figure 2:**
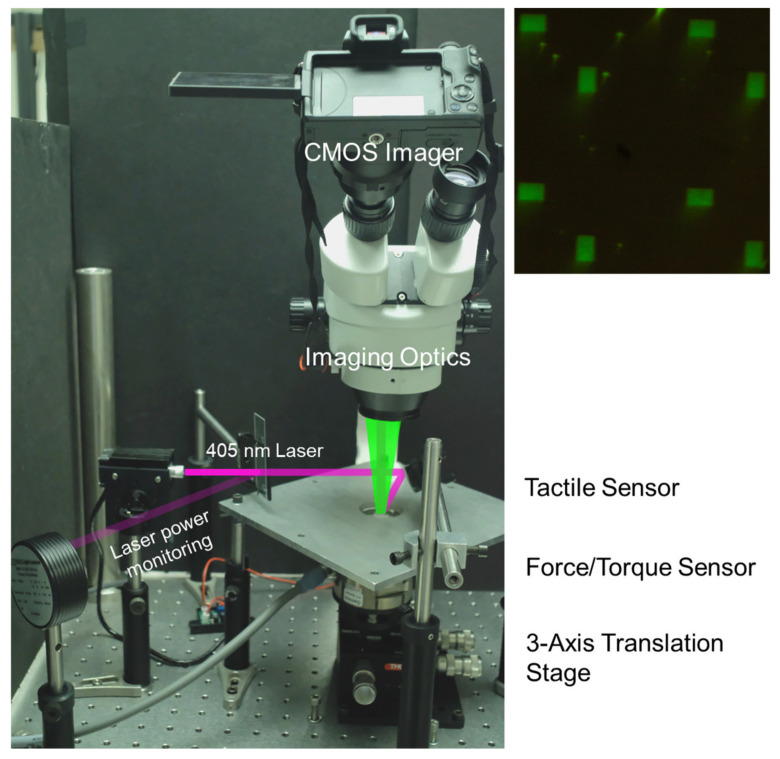
Schematic of the experimental setup consisting of a CMOS imager using a commercial DSLR camera and custom image optics, bias laser, optical power meter for monitoring the laser power, tactile sensor, and its holder (the square aluminum plate shown), and a 3-axis translation stage. The optical path of the laser is shown in purple and the emission of the tactile sensor is shown in green. The 425 nm long pass filter is housed within the imaging optics to remove the optical bias. During the measurements, the tactile sensor remained stationary while the force was applied by moving the testing surface rigidly mounted to the force/torque sensor using the translation stage. An example image collected by the CMOS imager is shown in the upper right. The green rectangles are the nanopillar arrays used for tactile sensing.

We designed the tactile sensor based on an array of nanopillar-shaped GaN LEDs as shown in the scanning electron microscope image in Figure 1a. Each nanopillar has an elliptical cross-section with dimensions of 337 nm × 171 nm. When an external shear force is applied to the tip of the nanopillar, the nanopillar is deformed, resulting in a redistribution of the electron and hole wavefunctions in the InGaN/GaN quantum wells as shown in Figure 1b, reducing the light emission intensity. Owing to the strong piezoelectric effect, the GaN nanopillar LEDs can function as a tactile sensor in combination with a photodetector array, such as a CMOS imager. Owing to the elliptical cross-section, the wavefunction redistribution responds asymmetrically to the applied shear forces. The sensor’s capability to measure the force’s direction is achieved by having two types of nanopillar arrays whose elliptical cross-sections are orthogonal to each other. Measuring the relative intensity changes of the two arrays simultaneously (see Figure 2) enables the reconstruction of a shear force at an arbitrary angle from orthogonal components parallel to the long and short axis of the elliptical cross-section and time evolution analysis. Previously, we have demonstrated the mapping of a two-dimensional shear force field. The force’s absolute magnitude was determined by comparing the measured relative intensity change with numerical simulations. The calculated intensity changes as a function of the force’s magnitude generated a lookup table to translate the measured intensity map to the force field [25]. In this work, we designed an experiment to directly measure the absolute magnitude of force applied to the tactile sensor, enabling the “lookup table” to be generated experimentally rather than numerically.

The experimental setup is shown in Figure 2. We used a commercially available force and torque (F/T) sensor (ATI Gamma), which has an accuracy of 0.01 N. The force and torque sensor acts as the supporting plane for a testing surface which directly contacts our tactile sensor. For this work, we chose two testing surfaces, a 500 µm thick rigid sapphire wafer coated with a thin photoresist (SPR 220 3.0, 2.2 µm thickness), and a flexible silicone rubber square. Each of these surfaces was attached rigidly to the sensing surface of the F/T sensor. The nanopillar tactile sensor was mounted with the nanopillars facing downward. It was optically biased [25] with a 405 nm laser for simplicity although an electrically biased version of the sensor was previously demonstrated [26]. The sensor response was recorded using a commercial DSLR camera equipped with a CMOS imager. A 425 nm long-pass optical filter was used to reject the biasing laser. Without the filter, the laser can saturate the imager’s detection pixels and lead to an inaccurate intensity measurement.

To apply the force, we kept the tactile sensor stationary while moving the F/T sensor with a three-axis translation stage. Contact between the testing surface and the tactile sensor was determined by monitoring the reported force normal to the supporting plane of the F/T sensor. Once the contact was firmly made, the testing surface was moved in the shear direction with respect to the tactile sensor. We moved the translation stage in the X direction with a 50 µm step size up to 500 µm. The X direction was aligned to one of the ellipse’s axes. At each displacement step, an image of the tactile sensor was captured with the CMOS imager, the F/T sensor data was collected, and a photodetector recorded the power of the biasing laser. We calculated the average green integer value reported by the CMOS imager for each visible array during each translation step. The green values were normalized against their respective arrays at the 0 µm translation image to determine the relative intensity emission of each nanopillar array. The intensity change is expected to be more sensitive when the force is parallel to the long axis.

## 3. Results

**Figure 3:**
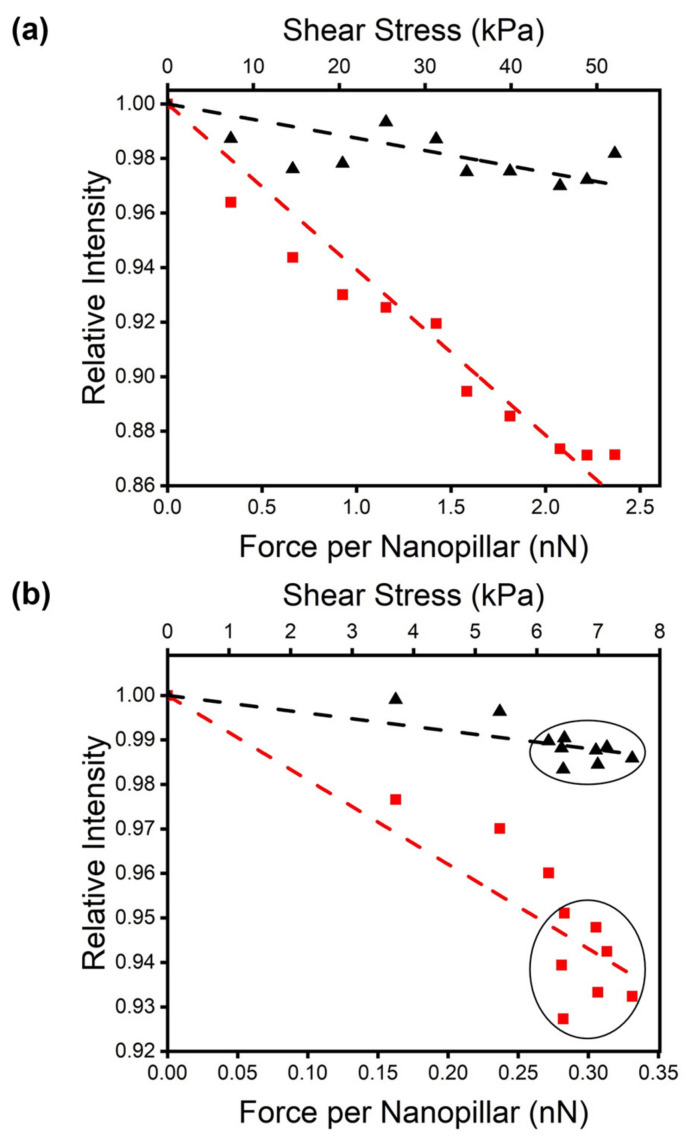
The response of the tactile sensor’s emission intensity versus a shear force applied to two testing surfaces: (**a**) SPR 220 photoresist and (**b**) silicone rubber. The force reading from the F/T sensor is converted to the shear stress (top axis) and force experienced by each nanopillar (bottom axis) based on the contact area of each testing surface with the tactile sensor. In each graph, the two curves were measured from the two nanopillar LEDs with their ellipse cross-section’s orientation orthogonal to each other. The black (red) curve corresponds to the LED with the ellipse’s short (long) axis parallel to the force. The dashed lines are the linear fits of the measurement data. In (**b**), the fluctuation of the measured intensity within the two circles suggested a slip between the testing surface and the tactile sensor.

Figure 3 shows the results. The two testing surfaces’ normal forces applied to the tactile sensor are 0.739 and 1.009 N for the photoresist and silicone rubber, respectively. The amount of normal force was chosen by observing the reading of the F/T sensor when a small shear force was simultaneously applied. We increased the normal force until the reading became stable and not noisy, which likely was an intrinsic limitation of the F/T sensor requiring a small but steady normal force in accurately measuring the shear force.

**Figure 4:**
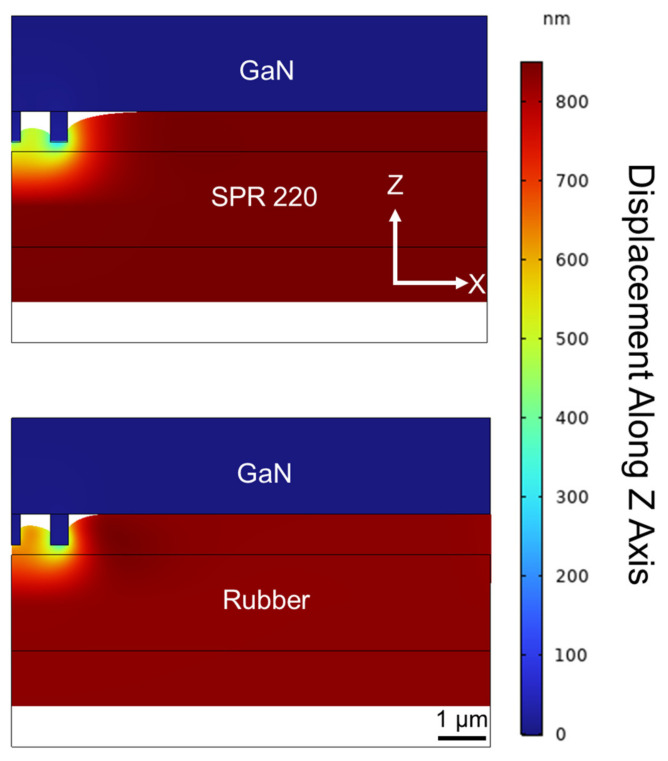
Simulation of testing surface’s compliance when in contact with the tactile sensor, which is shown upside down and modeled by two nanopillars on the left and a GaN substrate on the right. Only a normal force was applied. The coloring of the simulation corresponds to the vertical displacement of the simulation mesh. The maximum displacement was 850 nm, and the test surface started 200 nm away from the nanopillar’s tip. The top and bottom show the contact conditions of the SPR 220 photoresist and the silicone rubber, respectively.

Because of the considerable amount of normal force required during the measurements and the compliant nature of the testing surfaces, it is possible the testing surface was pushed into the nanopillars. Moreover, the tactile sensor used in the experiment did not have nanopillars covering the entire sample area. Part of the testing surface may contact the sample area without nanopillars, even if the sample area is 650 nm below the tip of the nanopillars. To better understand the interaction between the testing surfaces and the tactile sensor, we performed numerical simulations using COMSOL’s Solid Mechanics module to simulate the deformation of the testing surfaces when in contact with the tactile sensor. In the simulation, as shown in Figure 4, we created a 2 µm thick testing surface on a 2 µm thick sapphire substrate. The tactile sensor was modeled as two nanopillars with a height of 650 nm made of GaN on a 2 µm thick GaN substrate. Initially, the tip of the nanopillar was placed 200 nm away from the top of the testing surface. The top boundary of the GaN was constrained and not allowed to move. The bottom boundary of the testing surface underwent a prescribed displacement until the air gap was zero between the testing surface and the GaN substrate. The minimum normal force required to make contact was 0.212 and 0.140 N for the photoresist and silicone rubber, respectively. These forces are considerably smaller than the normal force applied by the testing surfaces in the experiment, suggesting that portions of both testing surfaces were in direct contact with the GaN surface of the tactile sensor, as shown in Figure 4.

**Figure 5:**
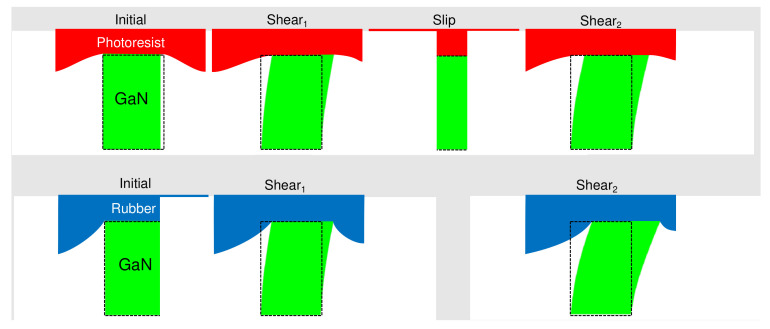
Schematic illustrating the light intensity from the nanopillar can start to fluctuate during the incipient slip. Without the slip (shown at the bottom panel), the nanopillar’s deformation continues to increase with an increasing translation of the testing surface (photoresist or rubber) with respect to the tactile sensor. When the incipient slip occurs (shown at the top panel), the nanopillar can snap back. As the contacting surface between the testing surface and the nanopillar is not perfectly flat (see Figure 4), the snapback can vary from nanopillar to nanopillar. Further translation will continue to deform the nanopillar until another incipient slip. This process leads to intensity fluctuation as shown by the data points within the circles in Figure 3. The displacement shown of green nanopillars above are scaled by a factor of 20,000 for clarity.

We calculated the shear stress applied during the measurements based on the simulation results. The contact areas for the photoresist and rubber are 51.7 and 8.722 mm^2^, respectively. We also show the amount of force each nanopillar experienced in Figure 3. The results agreed qualitatively and quantitatively with the previous numerical simulations on the nanopillar LED’s intensity’s dependence on the external force [25]. The rubber testing surface had more “friction” with the tactile sensor and was able to create larger shear stress. In contrast, the photoresist testing surface was extremely smooth. It could only remain gripped to the tactile sensor until the incipient slip began to occur at around 6 kPa, in both X and Y directions. When the photoresist testing surface continued to glide across the tactile sensor, the fluctuation of the tactile sensor reading was observed in Figure 3b, from both the X and Y sensors. The photoresist slips from the tip of the nanopillar until the latter can grab the resist again as illustrated in Figure 5. Microscopically, the resist is not perfectly flat as shown in Figure 4. Individual nanopillars do not always snap back to the same state each time. As a result, the slip leads to an intensity fluctuation which can be used to detect the incipient slip. Using a CMOS imager with a higher sensitivity can allow one to study the microscopic dynamics of the slip which will be investigated in future work. In contrast to the vision-based tactile sensor [4,5,6,7,8,9,10,11], our sensor’s observation of the slip is direct and does not require any post-measurement computation.

## 4. Conclusions

In summary, we demonstrated the direct measurement of a shear force with a calibrated magnitude using a GaN nanopillar LED-based tactile sensor. Both the magnitude and direction of the force were directly measured without the need to fit the data through a theoretical model or perform complex computations as in other vision-based tactile sensors. In other words, we experimentally validated the capability of the GaN tactile sensor to function as a force/torque sensor by itself. The torque measurement, while not directly demonstrated in this work, can be easily achieved with a multipoint measurement as has been shown previously [25]. All nanopillars are decoupled mechanically and electronically from each other. The change in emission intensity from each nanopillar is the result of a localized misalignment between the electron and hole wavefunctions. As a result, the mapping of the force field can be achieved with an extremely high spatial resolution, limited primarily to the resolution and sensitivity of the CMOS imager.

We also reported the capability of directly observing the incipient slip from the sensor reading without requiring any computation or prior assumptions of the testing surface’s characteristics. As the force applied by the testing surface on the tip of the nanopillar exceeds the friction between them, the nanopillar “snaps back” until the friction and the applied force are in balance. This behavior mimics our subconscious modulation of the force between two fingertips when grasping an object to prevent a slip. The results pave the way for using our sensor with robot proprioception and visual feedback to complete grasping, pose estimation, and item discovery tasks requiring robots to determine the size and shape of an applied shear force.

## Data Availability

The data presented in this study are available on request from the corresponding author.
